# Peripheral skin cooling during gravitational challenges in parabolic flight – experimental protocol, implementation, and case study of the CoolFly experiment

**DOI:** 10.3389/fphys.2025.1477311

**Published:** 2025-05-26

**Authors:** Tomas L. Bothe, Viktor Heinz, Niklas Pilz, Leon Fesseler, Andreas Patzak, Renana Bruckstein, Michael Nordine, Hanns-Christian Gunga, Oliver Opatz

**Affiliations:** ^1^ Charité – Universitätsmedizin Berlin, Institute of Physiology, Center for Space Medicine and Extreme Environments, Berlin, Germany; ^2^ Charité – Universitätsmedizin Berlin, Institute of Integrative Neuroanatomy, Center for Space Medicine and Extreme Environments, Berlin, Germany; ^3^ Department of Cardiology and Angiology, Hannover Medical School, Hannover, Germany; ^4^ Charité – Universitätsmedizin Berlin, Institute of Translational Physiology, Berlin, Germany; ^5^ Department of Anaesthesiology, Intensive Care Medicine and Pain Therapy, Goethe University Frankfurt, University Hospital Frankfurt, Frankfurt, Germany

**Keywords:** space, orthostatic in, cardiovascular stability, blood pressure, extreme environment, countermeasure

## Abstract

**Objective:**

Ensuring cardiovascular stability is critical for the lasting and prosperous success of human spaceflight. Astronauts are exposed to dynamic acceleration profiles and prolonged changes of gravity which pose serious acute and long-term health risks. Parabolic flight is a model for gravity induced cardiovascular instability. In this proof-of-concept, we aim at analyzing the feasibility and effectiveness of peripheral cooling (PC) as a countermeasure during parabolic.

**Methods:**

In this study, we employed a cross-over trial to investigate the effectiveness of PC in enhancing cardiovascular tolerance during gravitational changes simulated via parabolic flight. Continuous, non-invasive blood pressure, heart rate, peripheral oxygenation and brain oxygenation, peripheral blood flow, as well as skin and brain temperature were assessed. This study is a proof-of-concept for experimental feasibility and qualitative effectiveness of PC during parabolic flight.

**Results:**

Our case study data showed reductions in heart rate of 10.0% (6.79 bpm) and reduced changes in heart rate during gravitational changes (standard deviation 12.55 vs. 10.37 bpm). Further, we observed reduced blood pressure reactions to altered gravity (−20/+39 mmHg vs. −9/+8 mmHg), with minimal changes in skin (0.27°C) and brain core temperature (0.14°C) as well as reduced changes in micro-perfusion comparing PC with control.

**Conclusion:**

This proof-of-concept study demonstrates that peripheral cooling is feasible during parabolic flight and may attenuate cardiovascular responses, as indicated by reduced heart rate and blood pressure fluctuations. These preliminary findings support further controlled studies to assess PC as a non-invasive countermeasure to changes in gravitation.

## Introduction

Human life has evolved under Earth’s gravitational conditions. In consequence, human physiology is adapted to this environment, ensuring a constant and sufficiently oxygenated blood flow to all vital organ systems (Silver and Siperko, 2003). The gravitational force markedly determines fluid distribution within the body. This is particularly evident in instances of postural change, which can lead to alterations in cardiac pre- and after-load, and in the clinical manifestations of orthostatic hypertension (Klijn et al., 2015; Ricci et al., 2015).

Similar effects have been observed during human spaceflight. These include a marked caudal to cranial fluid shift when in microgravity and a prominent inverse redistribution after returning to planetary gravity (Hargens and Watenpaugh, 1996; Demontis et al., 2017). In response, the cardiovascular system detects an increased pre-load and actively downregulates cardiac chronotropy, inotropy, vascular resistance and blood volume to limit blood flow and pressure to the vital organs and prevent damage under the condition of microgravity (Tanaka et al., 2017; Gerber et al., 2018). Adaptation to these conditions leads to a severe cardiovascular deconditioning, represented by a decline in baroreceptor responsiveness, reduced cardiac reserve, relative hypovolemia and altered autoregulation. This culminates in post-flight orthostatic intolerance, experienced to various extents by all long-term space travellers (Blaber et al., 2011; Lee et al., 2015; Fu et al., 2019).

These changes are critical for the management of astronauts’ health after return to Earth. Furthermore, the diminished performance observed in the post-landing phase, which may potentially lead to orthostatic intolerance, is likely to constitute a significant challenge for future crewed missions aimed beyond low-Earth orbit (BLEO) (Baker et al., 2019; Moore et al., 2019). To ensure astronaut’s health, these new mission profiles demand safe, effective, and reliable countermeasures, that are not dependent on Earth-bound capabilities (Doarn et al., 2019; Scott et al., 2023). It is imperative that all countermeasures undergo a rigorous evaluation in spaceflight analogues prior to their implementation in space, such as parabolic flight.

Parabolic flight offers an excellent combination of rapid gravitational changes (hyper-gravity = 1.8 g, normo-gravity = 1.0 g, micro-gravity = 0.0 g) which induces acute physiological reactions that can be measured in real-time (Ploutz-Snyder, 2016; Cromwell et al., 2021). The richer and more robust the data produced in these contexts, the greater our assurance in the effectiveness of the suggested countermeasures. However, physiological monitoring during parabolic flight is highly complicated. The extreme, changing environment and short-term duration of gravitational states during parabolic flight necessitate careful selection of measurement paradigms. In cardiovascular monitoring, electrical signals (such as Electrocardiograms (ECG)) can be recorded comparably simply and reliably (Beckers et al., 2003; Verheyden et al., 2005). To the contrary, real-time measures of blood pressure (BP) are already highly complicated on the ground (Kario et al., 2024; Pilz et al., 2024). Multiple studies have provided data using finer-plethysmographic devices during parabolic flight (Schlegel et al., 1998; Limper et al., 2014). While feasible if conducted carefully, this technology is highly prone to measurement artefacts such as induced by changes in posture (gravitational effect) and temperature changes (vascular effect) (Wesseling, 1996; Romano et al., 2002). New measurement technologies (e.g., based on the pulse-transit-time) have been shown to provide comparably reliable and easily recordable BP measurements, even under highly dynamic physiological conditions (Pilz et al., 2022; Kario et al., 2024). Lastly, the combination of Laser-Doppler Flowmetry and parabolic flight proven near-infrared-spectroscopy provides a comprehensive measurement approach to monitor peripheral blood flow and oxygenation (Schneider et al., 2013; Shepherd and Öberg, 2013). The application of peripheral cooling (PC) further necessitates the monitoring of changes in body temperature.

We hypothesized that peripheral cooling (PC) could be an effective countermeasure to orthostatic intolerance, based on the physical response to cooling in form of a cutaneous vasoconstriction, increasing the total peripheral resistance and thereby after-load and arterial blood pressure (Charkoudian, 2010; Khoshnevis et al., 2015; Alba et al., 2019; Seeley et al., 2021). This effect of cold exposure has been described extensively in laboratory conditions (Durand et al., 2004; Korhonen, 2006). Clinically this is most impressively highlighted by the fact that cold winter temperatures lead to population wide increases in blood pressure (Stergiou et al., 2020). Beyond that, the number and severity of incidents of orthostatic intolerance is greatly amplified during the summer months, as the increased temperatures induce a peripheral vasodilation with subsequent blood pressure drop (Narkiewicz and Somers, 1998; Stergiou et al., 2015). It further already has provided promising results in improving orthostatic intolerance after multi-day head-down tilt bed rest, another Earth-bound spaceflight analogue (Keller et al., 2011).

Combining low-heat-conducting carbon-gel cooling pads with the TRPM8 activator menthol in a topical gel is a promising approach as it induces a strong cold sensation (critical for the induction of vasoconstriction and therefore potentially cardiovascular stabilisation) without meaningfully lowering core body temperature (Lee et al., 2012; Voronova et al., 2022). More precisely, the menthol induced vasoconstriction can lead to an increase of body temperature, counteracting the small temperature effect of carbon-gel cooling pads (Gillis et al., 2010).

This study assesses the effectiveness of PC as a countermeasure to orthostatic challenges during changes in gravitational force. The results presented here are based on a proof-of-concept analysis of the findings from the CoolFly experiment, which took place as part of the German Aerospace Agency’s (DLR) 39th parabolic flight campaign.

## Methods

### Participants and experimental location

The CoolFly experiment was conducted during the DLR’s 39th parabolic flight campaign, at Novespace in Merignac, France. A single subject (N = 1) has been included. The subject was a healthy, 34-year-old male with no prior micro- or hyper-gravity exposure. Exclusion criteria included any cardiac, pulmonary, neurological, or other system illness. Written informed consent was obtained from the subject before participation.

The experiment consisted of two parabolic flights, each conducted on a separate day. Each parabolic flight itself consists of 30 unique parabolas, flown in sets of five. After each parabola there is a period of steady flight of 1 minute. After each set of 5 parabolas, there is a 5-min break. After the first 3 sets of 5 parabolas each (halfway point), there is a longer pause of 8–12 min.

A parabola maneuver describes the plane pitching up from steady flight at full thrust to 47° for 20 s, creating 1.8 g (hyper-gravity) in the cabin. After the ascend, the thrust is set to minimum, allowing the plane to follow a parabolic trajectory, creating a micro-gravity (∼0 g) environment in the place for 22 s. In this process, the plane pitches down to 45°. To break the fall, the plane pulls up again for a period of 20 s, again creating an environment of 1.8 g.

To limit motion sickness during the parabolic flight, the subject was given an intra-muscular injection of Scopolamine by the medical team of Novespace on both flight days before boarding the airplane. Further, to mitigate the potential effects of scopolamine on the experimental results, the subject received a minimal intramuscular dose of 0.005 mg/kg Scopolamine 90 min before the onset of the parabolas. As cold temperatures abord the plane could induce physiological reactions affecting our experimental outcome, Novespace agreed to conduct this parabolic flight at 23°C (unlike the typical 17°C–20°C). Moreover, the participant wore a T-shirt during the control parabolas to protect him from potential wind-chill effects induced by the aircraft’s air conditioning.

### Experimental protocol

#### Experimental design

We evaluated the effectiveness of PC as a countermeasure to orthostatic stress during parabolic flight in a cross-over controlled case study design. To minimize the bias induced by the increased mental load during the first (excitement about first flight) and last set of five parabolas (getting ready for end of experiment), only data from the second until the fifth set of parabolas was analyzed for stress dependent parameters (e.g., heart rate). The subject was measured in a standing position during the full experiment and was advised to not use the muscle pump to actively counteract orthostatic effects. To homogenize the mental load and excitement state as much as possible, the subject was asked to perform psychometric tests (Flanker Test) (Sanders and Lamers, 2002) on a tablet computer during the parabolas.

#### Peripheral cooling

To induce a cutaneous vasoconstriction and thereby increase total peripheral resistance, afterload and blood pressure, we applied a combined PC countermeasure. As vasoconstriction is dependent on the activation of cold-sensitive TRP-channels, sensing a cooling of the skin surface temperature, and not on core-body energy drainage, we opted for a combination of carbon-gel cooling pads (Aubdool et al., 2014; Pan et al., 2018). These were fixated by cooling garments (CarbonCool, Global Healthcare SG, Singapore) to the lower extremity and abdomen and back and the application of a long-lasting menthol gel (BioFreeze, Reckitt Group, United Kingdom) (Charkoudian, 2010; Voronova et al., 2022). The carbon-gel cooling pads remain cold for a prolonged period, leading for a steady-state PC during the whole flight. The menthol gel additionally induced a cold-sensation by activating temperature-sensitive TRP channels and without further cooling down the participants (Bautista et al., 2007). The cooling garments and menthol gel were applied and attached to the test subject during the flight, immediately before the commencement of second 15 parabolas.

### Physiological measurement

#### Heart rate (Faros, SOMNOtouch NIBP, ABPMpro)

We recorded an electrocardiogram (ECG) in a full Holter configuration with three different devices. This was done to create redundancy and ensure maximum measurement quality for potential heart-rate variability analyses. The Faros 180 (Bittium, Finland) was used to record only the ECG. The Somnotouch NIBP and ABPMpro (both SOMNOmedics, Germany) devices were also used to record ECG data. The ABPMpro also offers preliminary impedance cardiography capabilities.

#### Blood pressure monitoring (SOMNOtouch NIBP, ABPMpro)

The two devices were also used to monitor the continuous and non-invasive blood pressure, relying on a pulse-wave-velocity measurement approach. The SOMNOtouch NIBP detects the pulse-arrival-time, derived from the ECG and a SpO_2_ finger clip (Bilo et al., 2015; Socrates et al., 2021; Bothe et al., 2023). The ABPMpro measures the pulse-transit-time (pulse-arrival-time corrected for cardiac pre-ejection period) from an ECG and a photoplethysmography sensor placed at the upper arm (Pilz et al., 2023). The device also offers a validated oscillometric, cuff-based blood pressure measurement capability which was used to calibrate both devices’ continuous measurement in between sets of *parabolas* to optimize measurement accuracy (Roth et al., 2023). For this, automated cuff-based blood pressure measurements were initiated with the device during steady flight. The cuff-based reference values were used to calibrate the continuous blood pressure estimation in a post-measurement analysis, as recommended by the device manufacturer. In addition, the ABPMpro detects changes in the gravitational force through its inbuilt accelerometer. Both devices were used synchronously to mitigate the known limitations of continuous blood pressure measurement (Pilz et al., 2022).

#### Peripheral perfusion

We monitored the peripheral perfusion using two different measurement approaches. The MoorVMS-LDF2 Laser Doppler (Moor Instruments, United Kingdom) was used to evaluate the peripheral blood flow and thereby vasoconstriction (Roeykens et al., 2016). The sensors were placed on the subject’s forearm and upper calf.

The NIRO 200 (Hamamatsu Photonics, Japan) near-infrared spectroscopy monitor was used to determine peripheral micro perfusion by monitoring the tissue oxygenation (Hyttel-Sorensen et al., 2011). The sensors were placed in close proximity to the Laser Doppler sensors on the subject’s forearm and upper calf.

#### Brain core and forehead skin temperature (T-Mini)

We non-invasively and continuously measured the forehead skin temperature and estimates of the core brain temperature using the T-Mini sensor (Drägerwerke, Germany). The sensor was attached to the subject’s forehead and fixated using a specifically designed headband. The data were recorded using the FUM-80 data recorder (KORA Industrie-Elektronik, Germany). The setup was successfully clinically tested in a surgical application before (Soehle et al., 2020; Janke et al., 2021).

#### Experimental monitoring setup

**FIGURE 1:**
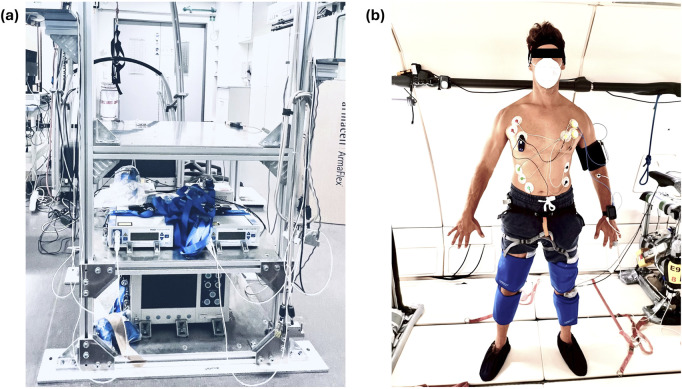
The left panel **(a)** shows the experimental rack. The data recording laptop was later attached to the top shelf computer, the Laser Doppler monitors (mid shelf) and the NIRO 200 near-infrared spectroscopy device (bottom shelf). The right panel **(b)** shows the measurement sensor placement displayed on a researcher who provided his consent for publishing the photograph. The attached sensors are the ECG sensors, the two continuous blood pressure monitors (subject’s left arm), the Laser Doppler and near-infrared monitors (subject’s left arm and calf), and the T-Mini sensor (head band). The PC garment (blue) is attached to the subject’s lower extremities. Additional cooling garments were placed around the subjects’ abdomen and lower back (not depicted).

The Faros ECG monitor, the SOMNOtouch NIBP and ABPMpro continuous blood pressure measurement devices, and the T-Mini core temperature sensor were attached directly to the subject. The devices were attached pre-flight and measurement was initiated as soon as the plane reached the experimental altitude before the onset of the parabolas. The MoorVMS-LDF2 Laser Doppler and NIRO 200 near-infrared spectroscopy devices were attached to an experimental rack fixated in the place. The sensors were attached to subject before the onset of the parabolas. The devices were connected to a laptop computer acting as central data recorder which was also connected to the rack. (Figure 1). Time synchronization between the devices was achieved by initializing all measurement with the data recording laptop.

### Data analysis and presentation

This work is designed as a methodological feasibility and implementation case study as well as a proof-of-concept for the effectiveness of PC as a countermeasure during parabolic flight. We therefore show preliminary case study (n = 1) data for each measurement approach and descriptively illustrate the observed effectiveness of PC. Data synchronization was achieved by initializing all measurements with the same data recording computer. This ensured a measurement synchronization of under one second.

### Ethics

Following article L. 1121-4 of the Public Health Code, this study was approved by a French Ethics Committee - Comité de Protection des Personnes Sud Méditerranée I (11/02/2022) and authorized by the French Competent Authority (04/01/2022). The experiment was conducted in agreement with the Declaration of Helsinki.

## Results

### Heart rate

The synchronized recording of acceleration and ECG data allows to analyse the connection between gravitational changes and changes in heart rate.

**FIGURE 2:**
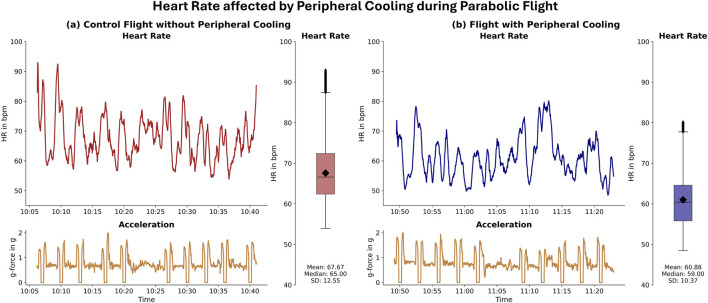
Heart Rate affected by Peripheral Cooling during Parabolic Flight: Difference in heart rate during the control and peripheral cooling part of the flight. The left panel **(a)** depicts the heart rate (upper part) and the acceleration trace (lower part) during 10 parabolas on the control part of the flight. The right panel **(b)** depicts the heart rate (upper part) and the acceleration trace (lower part) during 10 parabolas on the peripheral cooling part of flight. For both the control and the PC parts, boxplots are provided on the right side of each panel. For this case study subject, the mean heart rate and heart rate fluctuations during the parabolas were visibly reduced in the peripheral cooling condition.

Our case study data shows apparent changes in heart rate when comparing hyper-gravity and micro-gravity conditions. Each parabola induced an increase in heart rate during the hyper-gravity phase. During the subsequent micro-gravity phase, the heart rate recovered but did not reach baseline before rising again during the second hyper-gravity phase (Figure 2).

### Blood pressure monitoring

**FIGURE 3:**
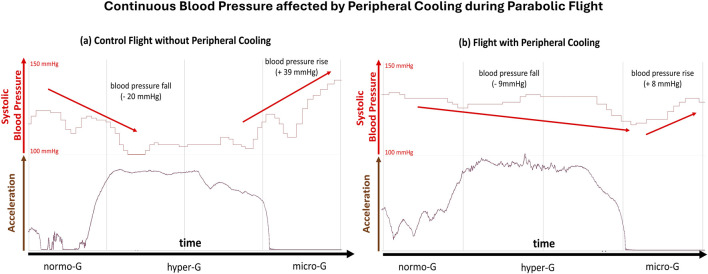
Continuous Blood Pressure affected by Peripheral Cooling during Parabolic Flight: Difference in continuous arterial blood pressure during the control and peripheral cooling flight parts of the case study subject. The left panel **(a)** depicts the systolic blood pressure (upper part, red) and the acceleration trace (lower part, brown) during a single parabola on the control part. The right panel **(b)** depicts the systolic blood pressure (upper part, red) and the acceleration trace (lower part, brown) during a single parabola on the peripheral cooling part. The baseline systolic pressure was increased during the part under peripheral cooling. Further, the blood pressure variability was visibly reduced during the peripheral cooling part, indicating a marked reduction in reaction to orthostatic challenge in the cooled condition.

The data recorded by the Somnotouch NIBP derived continuous estimates of continuous arterial blood pressure with beat-to-beat measurement intervals. Case study data for one parabola showed a blood pressure decrease during the hyper-gravity phase, followed by a greater increase in blood pressure during the subsequent micro-gravity phase (Figure 3).

### Peripheral perfusion

**FIGURE 4:**
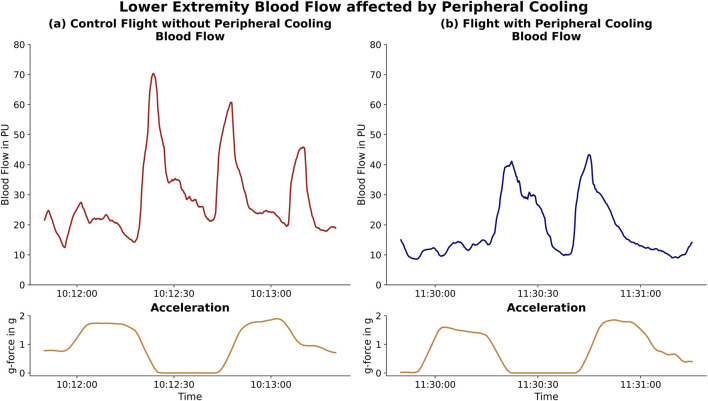
Lower Extremity Blood Flow affected by Peripheral Cooling: Difference in lower extremity blood flow during the changes in g-force (acceleration, lower panels, gold) within a parabola. The left panel [**(a)**, red] depicts the blood flow in the control part while the right panel [**(b)**, blue] shows the blood flow for the same subject during the cooled part. The decreased change in blood flow during the hyper-gravity phases and subsequent reduced drainage is depicted.

The Laser-Doppler measurement retrieved continuous and high-quality data of peripheral vascular flow. The data showed changes in peripheral blood flow coinciding with changes in gravitational force during the parabolas (Figure 4).

**FIGURE 5:**
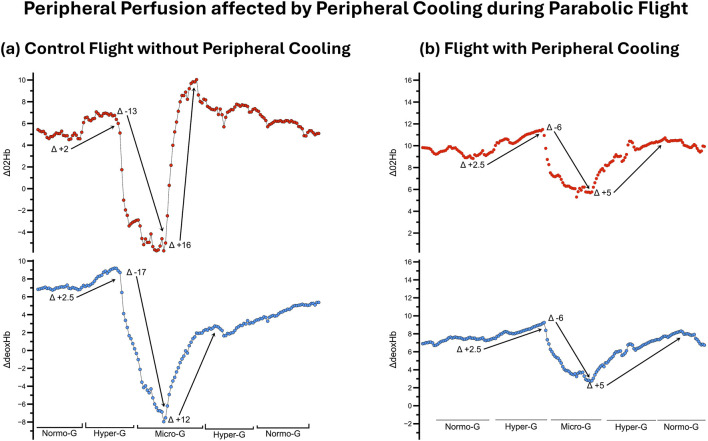
Peripheral Perfusion affected by Peripheral Cooling during Parabolic Flight: Difference in peripheral tissue oxygenation during the control and peripheral cooling flight part of the case study subject. The left panel **(a)** depicts the changes in oxygenated (upper part, red) and deoxygenated hemoglobin (lower part, blue) during a single parabola on the control part. The right panel **(b)** depicts the changes in oxygenated (upper part, red) and deoxygenated hemoglobin (lower part, blue) during a single parabola on the peripheral cooling part. Peripheral cooling visibly limits the variability of both the oxygenated and deoxygenated hemoglobin during changes in gravitational force, indicating a reduced change in perfusion during orthostatic challenge.

The available near-infrared spectroscopy data showed changes in tissue oxygenation during changes in gravitational force during parabolic flight (Figure 5). We experienced frequent measurement artefacts during the experiment.

### Skin and brain core temperature

**FIGURE 6:**
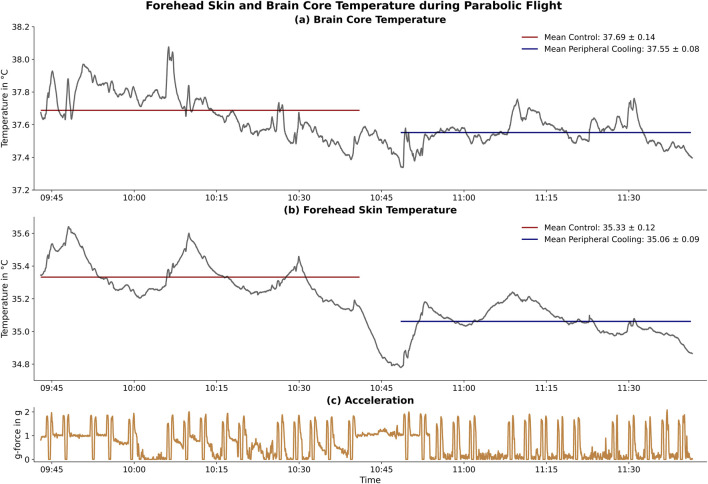
Forehead Skin and Brain Core Temperature during Parabolic Flight: Effect of peripheral cooling on the forehead skin and brain core temperature. The upper panel **(a)** shows the brain core temperature while the middle panel **(b)** shows the forehead skin temperature. The control (red) and the peripheral cooling (blue) parts and their respective mean temperature values are shown. The lower panel **(c)** shows the acceleration profile of all 30 parabolas flown in this flight. The time between the control and peripheral cooling part was used to apply the peripheral cooling countermeasure during steady flight. Little to no change in temperature occurs during the peripheral cooling phase.

The temperature measurement showed stable and high-resolution recordings of both forehead skin and brain core temperature. Our data showed differences below 0.3°C in both forehead skin and brain core temperature values comparing the PC and control (Figure 6).

## Discussion

One of the major challenges facing the ambitious future of human spaceflight, including reaching farther into space and incorporating a diverse astronaut corps, is to identify and validate reliable and effective countermeasures for orthostatic instability (Platts et al., 2014; Goswami et al., 2015; Lee et al., 2015; Jirak et al., 2022). The objective of this study was to evaluate PC as a potential candidate non-invasive, safe, and effective countermeasure to orthostatic challenge during parabolic flight. This proof-of-concept highlights the feasibility of PC as a countermeasure and offers single-subject descriptive results on its effectiveness.

In recent years, various countermeasures to cardiovascular instability have been investigated, such as intravenous isotonic fluids and oral salts (Vernikos and Convertino, 1994; Cowings et al., 2015), treatment with pharmaceutics such as Midodrine and Fludrocortisone (Ramsdell et al., 2001; Platts et al., 2004; Shi et al., 2004), compression garments (Platts et al., 2009), lower body negative pressure training (Watenpaugh et al., 2007), exposure to 1 g on a centrifuge (Moore et al., 2005; Goswami et al., 2015). However, most of these approaches suffer from either one or more severe drawbacks in regard to safety, non-invasiveness, applicability, their proven coutermeasuse effectiveness or their potential for long-term use. Studies have shown that while cuffs around the thighs can alleviate symptoms associated with fluid shifts, such as head congestion. But they do not significantly improve orthostatic tolerance post-flight (Robin et al., 2020). The combination of the TRPM8 activator menthol applied as a topical gel and cooling pads with low heat conductivity (carbon-gel) has not yet been studied during parabolic flight and may provide a flexible and effective alternative to existing countermeasures (Gillis et al., 2010; Shelhamer, 2016; Voronova et al., 2022).

We were able to record high-quality–high sample rate data with few artefacts–ECG, continuous blood pressure, peripheral blood flow and tissue oxygenation, preliminary impedance cardiography and skin forehead as well as brain core temperature. All measurements were non-invasive and provide high-frequency data, allowing us to analyse the effects of changes in gravitational force even in the quick changes between hyper-gravity and micro-gravity during parabolic flight. Further, the combination of used monitoring devices provided comprehensive data on changes in the cardiovascular system as well as high-quality temperature data, crucial for evaluating PC’s safety and effectiveness as a countermeasure. We therefore consider the measurement paradigms and devices used in this experiment as a useful physiological measurement toolkit that can be easily applied and adapted to other physiological and extreme environment studies.

Descriptively evaluating our data for singular subjects revealed a marked effect of PC on orthostatic tolerance in all measured cardiovascular parameters. Across the bench, PC decreased measures of variability and increased overall perfusion stability while decreasing cardiovascular load. This was indicated by a decrease in mean heart rate and its variability, an increase and stabilization in blood pressure and a decrease in peripheral flow and perfusion changes between hyper-gravity and micro-gravity. These changes are likely modulated by changes in overall autonomic nervous system reactions to parabolic flight and are beyond that influenced by individual and instantaneous factors like the breathing rate (Iwase et al., 1998; Iwase et al., 1999b).

This study is limited by its proof-of-concept and feasibility testing approach and does not represent a quantitative confirmation of the effectiveness of PC as a countermeasure. Further, research during parabolic flights is inherently limited by the short-term gravitational changes it induces, which prohibit to clearly separate whether the observed effects are fully due to the current gravitational state or lingering compensatory effects to the gravitational state just before (Shelhamer, 2016). The single-subject design does not allow for a randomization in the order of cooling and control flights, which would be needed to ensure that the effects of habituation and scopolamine are not affecting the overall results. Both sympathetic activation due to excitement as well as the effect of scopolamine, a parasympatholytic (anticholinergic) muscarinic receptor antagonist, are not expected to negatively affect the cardiovascular stability (Vybiral et al., 1990). Even small, potentially positive effects should be more pronounced in the first part of the flight (control), which further underlines the promising initial data on peripheral cooling. In spite of these limitations, our study revealed promising single-subject data on the effectiveness of PC, showcasing a reduction in parameter variability and therefore reaction to changes in gravitational state.

Our preliminary results validate the potential of PC as a countermeasure and create an impetus for further and quantitative research into the approach if cooling as a potential cardiovascular countermeasure to gravitational stress. The peripheral cooling demonstrated in our work does not meaningfully alter core body temperature. Further, the vascular mechanisms of thermoregulation are evolutionarily developed to act for full yearly seasons. Thus, peripheral cooling could offer an attractive tool to counteract orthostatic intolerance for hours, days, or weeks after the return from space to Earth or after landing on other celestial bodies in the future.

## Conclusion

This study demonstrates the feasibility of our experimental PC protocol as a cardiovascular countermeasure during parabolic flight. The obtained physiological parameters delivered high-resolution and reliable measurement data within the challenging confines of parabolic flight. This case study data showed that PC improved the orthostatic stability across the measured physiological parameters: it decreased heart rate while both increasing and stabilizing blood pressure as well as improving peripheral flow and oxygenation stability.

## Data Availability

The raw data supporting the conclusions of this article will be made available by the authors, without undue reservation.
